# AI-mediated writing and durable learning: a psychological theory of calibration, bypassing and uptake

**DOI:** 10.3389/fpsyg.2026.1866062

**Published:** 2026-09-28

**Authors:** Zihang Lan, Chen Niu

**Affiliations:** Faculty of Languages and Linguistics, University of Malaya, Kuala Lumpur, Malaysia

**Keywords:** cognitive bypassing, cognitive load, educational psychology, English as a foreign language writing, feedback uptake, generative artificial intelligence, metacognition, self-regulated learning

## Abstract

Artificial intelligence (AI)-mediated writing provides a critical case for educational psychology because it reveals how learners regulate cognition, effort, confidence, motivation, and beliefs about competence when fluent external support is available. In English as a foreign language (EFL) writing, such support can correct grammar, reformulate sentences, suggest organization, and sustain revision dialogs with learners who may not have immediate access to human feedback. These affordances invite a tempting inference: better AI-assisted drafts indicate better learning. This Hypothesis and Theory article qualifies that inference by proposing Calibration-Bypassing-Uptake theory, a psychologically grounded account of when AI-mediated writing support strengthens durable competence and when it mainly improves visible performance. The theory links five processes: perceived feedback affordances, dual calibration of source and self, cognitive-affective regulation, strategic uptake or cognitive bypassing, and delayed transfer. Calibration refers to learners’ ability to judge both the reliability of AI feedback and the adequacy of their own understanding. Cognitive bypassing refers to delegating a target learning operation to AI without substantively attempting, evaluating, or reconstructing it during the writing episode; its consequences for learning require separate testing. The article argues that AI supports EFL writing development when learners use feedback to predict, compare, explain, revise, and transfer, rather than merely accept improved text. It derives hypotheses about feedback type, cognitive load, learner proficiency, writing anxiety, self-efficacy, authorship beliefs, hybrid human-AI feedback, alongside a methodological proposition about measuring transfer. The central outcome is therefore not the polish of the assisted draft alone, but the learner’s later capacity to plan, justify, revise, and transfer with reduced support.

## 1 Introduction

Educational psychology has long been concerned with how learners regulate effort, confidence, emotion, and feedback use when visible performance does not necessarily indicate durable learning. This problem becomes sharper in artificial intelligence (AI)-mediated English as a foreign language (EFL) writing, where an external system can improve a draft before the learner has fully diagnosed the underlying difficulty. In this setting, learners now ask generative AI models to correct paragraphs, improve academic tone, diagnose grammar errors, translate tentative ideas into thesis statements, or compare alternative sentence versions (Barrot, 2023; Liu and Ma, 2024; Liu et al., 2025a; Liu Z. M. et al., 2026; Warschauer et al., 2023; Woo et al., 2024; Yan, 2023). The attraction is understandable. EFL writing is slow, uncertain, and emotionally exposed. It requires learners to plan content, retrieve vocabulary, manage syntax, monitor genre expectations, and revise under limited linguistic resources. AI assistance often appears exactly where difficulty becomes most visible: the moment when a learner knows that a sentence is weak but cannot yet repair it.

Recent syntheses show that this is no longer a marginal topic. Reviews of generative AI and second language (L2) written feedback report rapid growth in studies of writing quality, revision behavior, learner perceptions, assessment, feedback uptake, and AI literacy (Crosthwaite and Sun, 2026; Feng et al., 2025; Lee et al., 2026; Li, 2025; Xiao et al., 2026). A review of learner emotions in AI-assisted English as a second language (ESL) and EFL learning also identifies anxiety and enjoyment as recurrent variables in a field still concentrated in higher education (Zhang S. and Liu X., 2025). The evidence base is now large enough to move beyond the broad question of whether AI helps. The more psychologically important question is how learners convert AI feedback into durable knowledge, and when AI makes performance appear stronger while leaving learning fragile.

The problem is not that AI feedback is necessarily poor. A useful correction can still fail to become learning. A student may submit a more coherent essay after accepting several reformulations, but the improved product does not show by itself that the student can now plan an argument, detect a weak transition, choose an appropriate verb, or explain why a sentence needed revision. The task has been completed. Competence may or may not have changed. Educational psychology has long distinguished performance during support from durable learning after support is reduced (Soderstrom and Bjork, 2015). Generative AI intensifies this distinction because fluent performance support is now immediate, abundant, and conversational (Darvishi et al., 2024; Fan et al., 2025; Yan et al., 2025).

This article develops Calibration-Bypassing-Uptake theory. It integrates two mechanisms often treated separately. The first is calibration: learners must judge when AI feedback is reliable, when it fits the task, and whether they understand enough to accept, adapt, verify, or reject it. The second is cognitive bypassing: learners may use AI to skip operations that normally build writing competence, including retrieval, explanation, discrimination, error diagnosis, revision planning, and reflection. These mechanisms help explain why the same tool can scaffold learning for one learner and substitute for learning in another.

The article makes four contributions. It reframes AI-mediated EFL writing as a problem of self-regulated feedback processing rather than tool adoption alone. It identifies cognitive bypassing as a mechanism separating apparent fluency from durable learning. It treats calibrated trust as a metacognitive control process rather than a general attitude toward technology. Finally, it derives testable hypotheses and design principles for distinguishing helpful support from substitutive support. The aim is not to defend or reject AI. The sharper question is where the learner’s mind remains active when external language intelligence becomes abundant.

This framing also helps keep AI-mediated EFL writing inside the scope of educational psychology rather than treating it as a merely technological or pedagogical question. The object of analysis is not a platform, model, or classroom technique by itself. It is a learning situation in which external intelligence changes monitoring, effort allocation, source evaluation, self-efficacy, emotion, and transfer. The same visible practice, such as asking AI to revise a paragraph, may reflect careful self-regulation or near-total delegation. A psychological theory is therefore needed because product quality alone cannot reveal which process occurred.

### 1.1 Literature identification and selection

This article develops a selective theoretical synthesis rather than a systematic review. Literature identification drew on Web of Science and Scopus. The literature base spans AI-mediated EFL/L2 writing and written feedback in applied linguistics; self-regulated learning, metacognition, cognitive load, affect, and learning versus performance in educational psychology; and trust calibration, cognitive offloading, and agency in human-AI interaction. Selection is guided by relevance to a specified construct or proposed relationship, clarity of the study design and outcome measures, and applicability to writing or an explicitly identified mechanism. Foundational theoretical works are considered alongside recent empirical studies and reviews. Findings on assisted performance, learning processes, and delayed outcomes are distinguished, and evidence of benefits is considered alongside evidence of limitations. These principles define the scope of the synthesis without implying exhaustive coverage or a systematic screening protocol.

The cited studies provide evidence for individual mechanisms and the plausibility of their integration; they do not validate the complete Calibration-Bypassing-Uptake model. This article reports no original empirical test. The proposed paths, their ordering, and the distinctive contribution of cognitive bypassing therefore remain hypotheses to be tested directly, especially when evidence is drawn from contexts other than EFL writing.

## 2 EFL writing as a demanding educational-psychology task

EFL writing is not only a language-pedagogy activity. It is a demanding educational-psychology task in which memory, attention, monitoring, motivation, and affect must be coordinated while learners produce a public artifact that is evaluated by teachers, peers, institutions, and themselves (Hayes, 2012). The difficulty is not merely that learners know fewer words or make more grammatical errors. They must hold content, audience, genre, lexical choice, syntax, cohesion, and assessment criteria in mind while working through limited working memory.

Cognitive load theory helps explain this difficulty. Learning depends partly on how limited cognitive resources are allocated (Sweller et al., 2011; de Bruin et al., 2020). In EFL writing, intrinsic load arises from coordinating ideas and linguistic form. Extraneous load comes from avoidable obstacles such as unclear tasks, confusing feedback, or too many unprioritized comments. Germane processing is effort directed toward schema construction, comparison, explanation, and transfer. AI can reduce load by clarifying an error or offering a model sentence. It can also increase load by producing too many alternatives, requiring prompt management, or forcing learners to evaluate fluent but uncertain output. Working memory research in technology-enhanced language learning and classroom work on ChatGPT-assisted writing both suggest that AI changes the distribution of effort rather than simply lowering it (Deng, 2026; Woo et al., 2024).

A recent scale for AI-assisted L2 writing makes this redistribution visible. Yao and Fan (2025) identify prompt management, critical evaluation, integrative synthesis, and authorial core processing as key dimensions of cognitive demand. The classification matters because AI use is not automatically lighter work. A learner who compares three thesis statements and explains why one fits the argument is still doing authorial work. A learner who asks AI to generate the thesis, outline, and concluding claim before any self-generated attempt may complete the task while doing less of the work that strengthens independent writing. Prompt studies similarly show that interaction with ChatGPT can become part of composing itself, not a neutral add-on (Hwang M. et al., 2025).

Emotion is equally central. L2 writing exposes gaps in competence and invites correction. Research on foreign language emotions shows that anxiety and enjoyment can coexist rather than simply replacing one another (Dewaele and MacIntyre, 2014; Yu, 2025). Skill-specific L2 writing research also indicates that boredom is intertwined with writing motivation and boredom-coping, reinforcing the need to treat affect and motivation as part of the writing process (Solhi E. et al., 2026; Solhi D. et al., 2026). Studies of L2 writing also link cognitive load, anxiety, self-efficacy, and performance, suggesting that cognitive and affective demands should be analyzed together (Shen and Tao, 2025; Wang et al., 2024; Zhang S. and Liu X., 2025). AI can change this climate by offering private, repeated, low-stakes feedback. That can support persistence. Yet reduced anxiety is educationally beneficial only when it frees attention for monitoring and experimentation. If relief comes mainly from letting the system decide, comfort may conceal dependency.

Motivation and self-beliefs add a further layer. Self-efficacy concerns belief in one’s capacity to organize and execute actions needed for performance (Bandura, 1997). Self-determination theory emphasizes autonomy, competence, and relatedness as conditions for high-quality motivation (Ryan and Deci, 2017). Recent AI-language-learning studies connect AI use with motivation, engagement, self-efficacy, enjoyment, and self-regulated learning (Huang and Mizumoto, 2025; Liu Z. M. et al., 2026; Wang, 2025; Wang and Wang, 2024; Wang et al., 2025). AI-IDLE research further links generative AI use beyond formal instruction with enjoyment and motivational self-concept (Liu G. L. et al., 2024; Liu et al., 2025d), and skill-specific AI-IDLE writing research connects L2 writing future selves and writing enjoyment with AI-mediated informal writing activity (Zou M. et al., 2025). These effects matter because helpless learners often avoid revision. Yet confidence becomes adaptive only when it remains linked to actual capability. An AI-polished draft can inflate perceived competence if learners attribute external fluency to personal mastery.

Feedback research explains why these distinctions matter. Feedback is powerful not because information exists, but because learners interpret and use it. Butler and Winne (1995) framed feedback as inherent to self-regulated learning because monitoring and control depend on information about performance. Hattie and Timperley (2007) showed that feedback can address task, process, self-regulation, or self levels, with different learning consequences. Later work on feedback literacy emphasizes that uptake requires evaluative judgment, agency, and opportunity for action (Carless and Boud, 2018; Carless and Winstone, 2023; Lipnevich and Panadero, 2021; Panadero and Lipnevich, 2022). L2 writing and automated-feedback research reaches the same conclusion: digital or teacher feedback becomes educational only through affective, behavioral, and cognitive engagement (Deeva et al., 2021; Koltovskaia, 2020; Mao and Lee, 2024; Pearson, 2024; Yan and Zhang, 2024). AI enters this feedback ecology with an added complication. It is fast, abundant, fluent, and often authoritative in tone even when the advice is mismatched or wrong (Lan et al., 2026).

The central psychological challenge is temporal. During drafting, AI may make learners feel competent because the text becomes better; after support is removed, the relevant question is whether learners can recover the reasons behind the improvement. A learner may remember that the model changed a sentence, but not the rule, genre move, or rhetorical criterion that justified the change. Durable development therefore requires more than exposure to correct language. It requires opportunities to encode reasons, test them, and reapply them under altered conditions. This is why AI-mediated writing should be studied through self-regulation, metacognition, and transfer rather than through product scores alone.

## 3 Performance support and durable learning

The distinction between performance and learning is the hinge of the theory. Performance is what learners can do during or immediately after support. Learning is inferred from later, flexible, less-supported use (Soderstrom and Bjork, 2015). Generative AI can quickly improve performance: it reformulates sentences, removes errors, reorganizes paragraphs, and generates examples. These changes may be useful, but they become misleading if the improved artifact is treated as direct evidence of improved competence.

Consider a learner who writes, “online classes make students convenient because they can study at home.” AI may suggest, online classes are convenient because students can study from home. If the learner notices that “convenient” describes classes rather than students, explains the pattern, and later applies it in a new sentence, feedback has supported learning. If the learner merely replaces the sentence, the draft improves while the underlying knowledge may remain unchanged. Both outcomes look similar in the revised text.

The same problem extends to higher-order writing. A learner may ask AI to make a paragraph more critical. The model adds a counterargument about unequal internet access and reduced peer interaction. The paragraph now appears stronger, yet the learner may not understand why contrast improves the argument or how to generate comparable moves later. The issue is not whether the AI suggestion is good. It is whether the learner engages in the cognitive work that would make the suggestion transferable.

Recent evidence makes this concern concrete. Yan et al. (2025) argue that generative AI can create performance gains without the deeper cognitive and metacognitive processing required for learning. Fan et al. (2025) similarly found that ChatGPT support improved essay-score gains, while knowledge gain and transfer did not show the same advantage. Older learning research anticipated this pattern. Conditions that feel smooth in the moment often produce weaker durable learning than effortful retrieval, generation, spacing, and variation (Bjork and Bjork, 2020; Karpicke and Blunt, 2011; Roediger and Karpicke, 2006). AI creates a new version of this old problem: it can reduce unnecessary difficulty, but it can also remove the struggle through which schemas are built.

A measurement trap follows. Assisted draft quality is tempting because it is visible, scoreable, and often improved by AI. Yet it may index the quality of the external support more than the learner’s developing capacity. The same score gain can arise from explanation-based learning, unprocessed correction, motivational persistence, better prompting, or wholesale substitution. A high-quality theory must therefore ask not only whether the text improved, but what operation changed inside the learner: monitoring accuracy, retrieval strength, genre judgment, error diagnosis, authorial control, or none of these.

This distinction explains why simple AI-versus-no-AI studies are insufficient. If an AI-supported group produces better revised drafts, several mechanisms remain possible. Learners may have internalized revision strategies. They may have accepted accurate corrections without understanding them. They may have become more motivated, learned to prompt rather than write, or developed false confidence. Without delayed writing, confidence judgments, revision explanations, and process traces, the researcher cannot know whether the outcome reflects learning or performance support.

Feedback type can shift the balance. Direct correction and full reformulation may produce rapid product gains. Explanatory, contrastive, and question-driven feedback may produce slower immediate improvement but stronger transfer because learners must infer, compare, and justify (Lukešová and Jennings, 2026). Studies comparing AI feedback, teacher feedback, human feedback, automated writing evaluation, and hybrid feedback increasingly show that form, timing, and engagement cannot be reduced to correction accuracy alone (Guo et al., 2024; Koltovskaia et al., 2024; Lin and Crosthwaite, 2024; Lu et al., 2024; Ma et al., 2026; Muñoz Muñoz et al., 2025; Zou S. et al., 2026). The most learning-supportive AI feedback may therefore be less efficient at producing instant polish because it preserves enough friction for the learner to remain cognitively present.

## 4 Cognitive bypassing as a psychological mechanism

Cognitive bypassing refers to delegating a target learning operation to AI without substantively attempting, evaluating, or reconstructing that operation during the writing episode. The target operation, such as diagnosing an error or planning an argument, must be specified before observing performance. This is a process definition: weaker subsequent knowledge, judgment, or revision skill is a predicted consequence, not a criterion for identifying bypassing. It is narrower than repeated tool dependence and concerns which learning operation is displaced, even when the AI output is accurate.

The bypassed operations are not peripheral. In EFL writing they include retrieving vocabulary, forming tentative meanings, choosing grammatical options, diagnosing errors, explaining revisions, planning paragraph logic, judging genre fit, and reflecting on what should transfer to the next text. These operations are effortful because learners coordinate incomplete knowledge under uncertainty. They are also the operations through which writing competence develops. AI becomes risky when it performs them in ways that leave the learner mainly managing the interface.

Bypassing can occur at different grain sizes. At the word level, a learner may repeatedly accept lexical substitutions without examining collocation or register. At the sentence level, the learner may replace awkward syntax without understanding the form-function relation. At the paragraph level, AI may supply topic sentences, transitions, and counterarguments that conceal weak rhetorical planning. At the task level, the learner may outsource brainstorming, organization, revision, and final polishing. Each act may seem minor, but repeated delegation can reorganize the learner’s writing routine around obtaining fluent output rather than building control.

This mechanism differs from beneficial cognitive offloading. Writers have always used external supports: dictionaries, corpora, rubrics, exemplars, peer comments, grammar handbooks, and teacher feedback. Educational psychology does not require unaided struggle for its own sake. The key distinction is whether support removes unnecessary barriers or removes the core learning operation. Research on cognitive offloading shows that external tools can extend cognition while changing what is stored, monitored, and retrieved internally (Gilbert et al., 2023; Iqbal et al., 2025; Richmond and Taylor, 2025). A dictionary supports lexical choice if the learner still evaluates meaning and use. A complete AI paragraph may eliminate the need to retrieve, test, and organize language. One support helps the learner walk a route; the other teleports the learner to the destination (Table 1).

**Table 1:** Cognitive bypassing and related constructs.

Construct	Definition and mechanism	Observable behavior	Expected learning implication
Cognitive bypassing	AI displaces a specified learning operation without substantive learner processing during the episode.	The learner delegates diagnosis or planning and neither evaluates nor reconstructs it despite a suitable opportunity.	Weaker later control is predicted and tested separately; correct AI output can still involve bypassing.
Cognitive offloading	External resources reduce internal task demands; they need not displace the target learning operation (Gilbert et al., 2023).	A writer uses a dictionary or external notes while retaining evaluation and explanation.	Performance may improve; learning depends on the operation retained or displaced.
Shallow processing	Limited semantic elaboration of material, whether or not an external agent performs the task (Craik and Lockhart, 1972).	A writer copies or attends to surface form without explaining its meaning or function.	Retention may be fragile; AI delegation is not necessary.
Automation overreliance	Reliance exceeds what the quality of automated advice warrants (Buçinca et al., 2021).	A writer accepts inaccurate or inappropriate advice despite reasons to question it.	Decision quality is at risk; correct reliance does not establish that learning occurred.
Reduced learner agency	Diminished control over goals and actions within a learning activity (Darvishi et al., 2024).	The learner allows the system to set goals or make choices with little self-directed judgment.	Self-regulation may weaken; agency can also be constrained without displacing a particular cognitive operation.

Self-regulated learning theory shows how bypassing can occur across a writing cycle. In Zimmerman’s model, learners move through forethought, performance, and self-reflection (Zimmerman, 2002). During forethought, AI can bypass goal setting when learners request an outline before forming even a rough plan. During performance, it can shift monitoring from meaning and structure to prompt management. During reflection, it can blur attribution because the final draft is co-produced and the learner cannot tell which improvements came from personal understanding. Recent evidence that higher-performing learners show more coordinated self-regulated patterns during generative AI-assisted writing reinforces the need to analyze regulation, not merely tool access (Jia et al., 2026).

These constructs can overlap within an episode. Bypassing adds an operation-specific claim about where AI substitutes for learner processing; it is not a claim that shallow processing or offloading is newly discovered. Its added value depends on whether process-based measures distinguish this substitution from the related constructs and improve prediction of later learning beyond them.

The risk is subtle because activity can look engaged. A learner may spend an hour prompting AI, comparing outputs, and revising repeatedly. Behaviorally, this appears active. Cognitively, it may still be shallow if the goal is only to obtain a better version. More prompts do not automatically mean more learning. Longer interaction does not automatically mean deeper processing. Process data must therefore distinguish explanation, diagnosis, and reconstruction during use from fluent text curation.

Cognitive bypassing is strengthened by metacognitive illusion. People often feel they understand systems until they must explain them in detail (Rozenblit and Keil, 2002). AI writing can create a comparable illusion: learners recognize a better sentence, select it, and feel ownership over the improved draft without being able to produce a similar sentence later. Recognition is mistaken for production. Exposure is mistaken for understanding. Adoption is mistaken for authorship.

AI also complicates source evaluation. Human-AI decision research shows that trust should be calibrated to system reliability, and that confidence cues or explanations can increase reliance even when accuracy does not improve (Jacovi et al., 2021; Lee et al., 2025; Zhang et al., 2020). Cognitive forcing functions can reduce overreliance by requiring users to pause, inspect, and justify AI-supported decisions (Buçinca et al., 2021). Large language models may also be poorly calibrated about their own uncertainty; evidence from medical reasoning shows that fluent systems can answer confidently while failing to recognize unknowns (Griot et al., 2025). The domain differs from EFL writing, but the lesson is relevant: fluent tone is not evidence of reliable metacognition in the source.

Bypassing is more likely when AI is used before a self-generated attempt, when feedback is global and fully rewritten, when assessment rewards polish more than reasoning, when learners lack criteria for judging output, or when institutional rules make legitimacy and authorship opaque (Dang and Wang, 2024; Jung et al., 2026; Zhai et al., 2024). It is not inevitable. Advanced learners may use AI to test alternatives while retaining control, and anxious learners may need AI to lower threat before revision becomes possible. The proposed mechanism is conditional: learning may be weakened when task structure and learner appraisals turn external fluency into a substitute for internal processing.

## 5 Calibration as the control system of AI-mediated writing

Calibration is the process by which learners align confidence, trust, and control decisions with actual knowledge and feedback quality. In AI-mediated writing, calibration has two targets. Source calibration concerns the learner’s judgment of AI feedback: is this suggestion accurate, relevant, genre-appropriate, and worth using? Self-calibration concerns the learner’s judgment of personal understanding: do I know enough to accept, adapt, ask for explanation, or reject it? This account builds on metacognitive monitoring, in which learners evaluate cognitive states and use those evaluations to guide action (Flavell, 1979; Lee et al., 2025).

The two judgments are intertwined. Learners who underestimate themselves may defer too quickly to AI. Learners who overestimate themselves may reject useful feedback or stop seeking explanation too early. Learners who overtrust AI may accept plausible but inappropriate reformulations. Learners who distrust AI indiscriminately may lose useful scaffolding. Learning depends on the fit among task demands, learner knowledge, and source reliability. Calibration is the control system that manages this fit.

Calibrated trust should not be reduced to a favorable attitude toward AI. In writing, learners need item-level and task-level trust. A model may be reliable for article use in a simple sentence and unreliable for a disciplinary theoretical claim. It may improve grammar while weakening stance. Recent evidence suggests that large language model (LLM)-generated academic writing can use a narrower range of stance and engagement features than human writing, which means polish may coexist with reduced rhetorical nuance (Mo and Crosthwaite, 2025). Calibrated trust, therefore, means knowing when the system is likely to help with the present problem and what verification is needed.

Five appraisals shape calibration. Reliability concerns whether feedback is likely to be correct. Relevance concerns fit with task, genre, audience, and writer goal. Controllability concerns whether learners can act on feedback without surrendering authorship. Cost concerns the effort required to understand, verify, and adapt the suggestion. Learning value concerns whether feedback helps learners do something better later. In assessment contexts, calibration also concerns whether AI-generated scores align with teacher criteria rather than merely appear consistent or authoritative (Alkhofi, 2026). These appraisals are cognitive, motivational, and affective at once.

For EFL learners, these appraisals are unevenly distributed across linguistic levels. A learner may calibrate grammar feedback reasonably well because the rule is familiar, but be poorly calibrated about argument structure, stance, citation framing, or disciplinary voice. Conversely, a learner may distrust AI grammar suggestions after seeing one error while still over-relying on its global organization. Calibration is therefore not a stable trait applied uniformly to all feedback. It is a situated judgment that varies across task, genre, source, and the learner’s own developing knowledge.

Authorship requires special attention because generative AI blurs the boundary between feedback and co-authorship. A teacher’s comment usually points to a problem; a model can produce the replacement. Learners then face a psychological and ethical question: is this still my sentence? Students’ decisions about whether to ask ChatGPT or a teacher are shaped by convenience, authority, nuance, evaluation, cultural appropriateness, and authorship concerns (Yin, 2025). Work on voice and critical AI literacy in L2 writing likewise suggests that AI can flatten stylistic individuality unless learners can position themselves, evaluate output, and explain how suggestions relate to their goals (Darvin, 2025; Sandstead and Kibler, 2025; Wang and Wang, 2025). A related digital-twin proposal separates machine execution from human responsibility (Lan and Luo, 2026).

Calibration also affects cognitive load. Poor calibration increases extraneous load because learners must manage uncertainty without criteria. Overly broad AI feedback creates option overload. Under-calibrated learners may verify everything inefficiently or verify nothing. Better design can reduce unnecessary search while preserving meaningful evaluation. For example, visualized generative AI feedback has been shown to influence revision quality, emotions, and cognitive load, suggesting that presentation format shapes both affective experience and processing (Zou B. et al., 2025). The key issue is not whether AI use is effortful. It is whether effort is allocated to prompt management alone or to critical evaluation, synthesis, and authorial processing.

Emotion changes calibration. Anxiety can narrow attention and push learners toward quick closure. Enjoyment can sustain exploration but also make interaction feel smooth enough that learners stop checking. AI may lower the threat of public correction, but high-stakes use may increase anxiety when students worry about detection, legitimacy, or loss of authorship. Studies of AI-supported language learning show that affective benefits should be interpreted together with regulation and engagement rather than treated as standalone outcomes (Wang and Wang, 2024; Wang et al., 2025; Xiao et al., 2024; Zhang S. and Liu X., 2025). The most productive condition is not simple comfort. It is low threat with sustained responsibility.

Self-efficacy follows the same logic. AI can build adaptive self-efficacy when learners experience themselves improving through guided effort. It can create false self-efficacy when learners attribute AI performance to personal skill. After AI feedback, can the learner explain what changed, revise a similar sentence without help, or predict which part of the next paragraph needs attention? Research on AI literacy, AI competence, and self-regulated learning makes this distinction important because confidence in AI-supported work may not track independent competence (Bećirović et al., 2025; Delcker et al., 2024; Huang and Mizumoto, 2025; Shen and Tao, 2025). Educationally, the opposite of overreliance is not avoidance. It is calibrated participation.

## 6 Calibration-Bypassing-Uptake theory

Calibration-Bypassing-Uptake theory starts from a simple premise: external feedback becomes learning only when learners convert it into internal regulation. Generative AI changes the form of feedback, not this principle. It makes feedback faster, more plentiful, conversational, and capable of producing replacement text. It also makes feedback harder to evaluate because the source is fluent, opaque, and variable. The theory models AI-mediated writing development through five linked processes.

Figure 1 visualizes the proposed Calibration-Bypassing-Uptake model by showing how perceived AI feedback affordances pass through dual calibration and cognitive-affective regulation before diverging into strategic uptake or cognitive bypassing, with delayed transfer serving as the criterion for durable learning.

**Figure 1:**
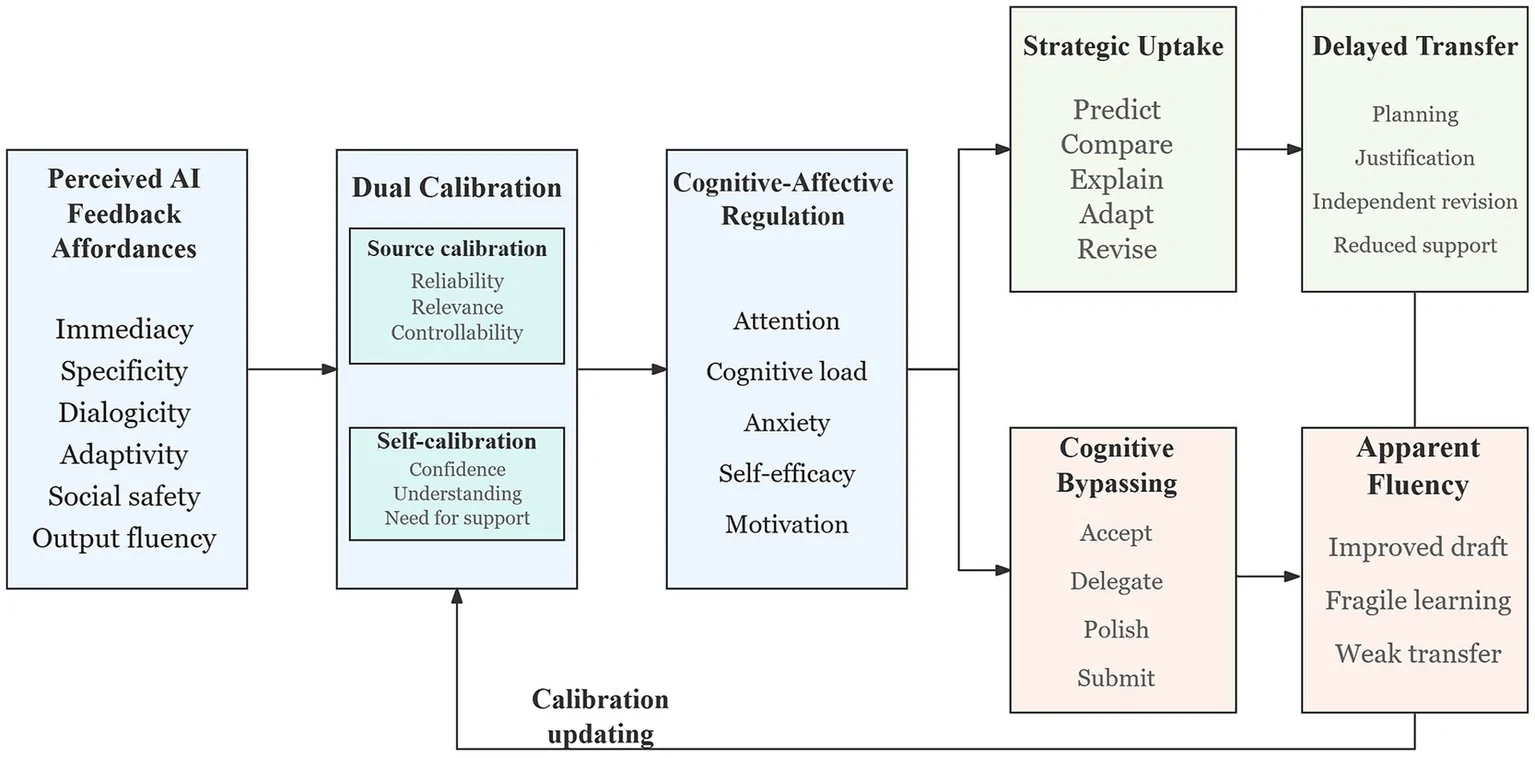
Calibration-Bypassing-Uptake theory of AI-mediated EFL writing.

The first process is perceived feedback affordance. Learners do not respond to AI as a neutral system. They respond to perceived possibilities and risks: immediacy, specificity, dialogicity, adaptivity, social safety, and output fluency. For a high-anxiety learner, social safety may matter most. For a high-proficiency learner, rapid comparison of alternatives may be more valuable. For a low-proficiency learner, the same output may appear useful but hard to evaluate. Feedback-seeking ability also matters because learners’ prompts partly determine what the system can provide (Yan, 2025).

The second process is dual calibration. When feedback appears, learners judge both the source and themselves. Is the suggested tense correct? Does the revision fit academic style? Has meaning changed? At the same time, the learner judges personal understanding. Is the original error clear? Can the rule be explained? Is further help needed? These judgments are fast, partial, and emotionally colored, but they drive control decisions. The same AI suggestion can lead one learner to strategic uptake and another to bypassing.

Within an episode, perceived feedback affordances are antecedents. Dual calibration precedes two distinguishable regulation pathways, which precede strategic uptake or bypassing; these process variables mediate the proposed relationship with later transfer. Proficiency and baseline feedback literacy are antecedent learner resources, while baseline writing anxiety is an antecedent risk factor. Feedback literacy and experimentally provided calibration scaffolds are proposed moderators of the proficiency-to-bypassing and anxiety-to-bypassing relationships (H6). Baseline self-efficacy informs initial self-appraisal and is measured separately from episode-specific confidence accuracy. Teacher guidance is an instructional antecedent intended to improve calibration (H8), rather than a benefit attributed to teacher presence alone. Changes in anxiety, persistence, and self-efficacy during an episode are potential responses to support, not interchangeable baseline moderators. Assisted text quality and delayed transfer are separate outcomes. Roles thus depend on measurement timing; baseline and post-feedback scores of the same construct must not be treated as the same variable.

The third process, cognitive-affective regulation, is an umbrella for two related pathways rather than a single latent variable. Cognitive regulation concerns allocating attention, managing extraneous demand, and selecting comparison or monitoring strategies; cognitive load is a demand state, not itself a regulatory action. Affective-motivational regulation concerns managing threat, sustaining effort, and maintaining control. Anxiety and enjoyment are affective states, self-efficacy is a competence belief, and autonomy is perceived control; their changes and associated regulation strategies should be measured separately. The two pathways may operate in parallel between calibration and subsequent uptake and may covary, without being added into one undifferentiated score. Calibration is assessed before processing, regulation during processing, and uptake at the feedback decision. Across episodes, outcomes can update later appraisals, so the within-episode ordering does not exclude longer-term feedback loops.

The fourth process is the fork between strategic uptake and cognitive bypassing. Strategic uptake occurs when learners interpret, evaluate, adapt, and integrate feedback with a substantive rationale linked to the target operation. It is not identical to acceptance. A learner may strategically reject AI feedback after noticing that it changes meaning or violates genre expectations. Cognitive bypassing occurs when learners delegate the operation without adequate processing. Studies of teacher and ChatGPT feedback show why this distinction matters: learners may use AI feedback productively for some issues but engage more deeply with teacher feedback in others (Lin and Crosthwaite, 2024; Zou S. et al., 2026). The acceptance behaviors and apparent-fluency outcomes shown in Figure 1 illustrate possible pathways, not diagnostic criteria: acceptance can be strategic, and weak transfer does not establish bypassing.

The fifth process is delayed transfer, assessed through three distinct outcomes: unaided performance on a new task, performance with reduced or bounded support, and more selective AI-supported performance under comparable tool access. These test internalized skill, performance as scaffolding fades, and judgment in an AI-rich environment, respectively. For the third outcome, improvement must include more accurate source evaluation, justified acceptance or rejection, or adaptation to a changed genre or tool, rather than polish alone. Failure on an unaided task limits a claim about independent skill; it does not by itself negate gains in skilled AI use. The three outcomes should be reported separately.

Two learners may receive the same suggestion to replace a vague claim with a sharper topic sentence. Learner A accepts the new sentence, asks AI to improve the paragraph, and submits the polished version. Learner B compares original and revised sentences, identifies the added causal relation, rewrites another topic sentence independently, and later checks whether the same move works in a new paragraph. Learner A may produce the stronger immediate draft. Learner B has engaged in comparison, explanation, self-testing, and transfer. The difference is psychological, not technological.

The theory also explains why hybrid human-AI feedback can be valuable. AI can provide speed, examples, and repeated low-stakes practice. Teachers can support calibration by asking learners to justify revisions, identify useful suggestions, and detect when AI changes meaning. Studies of teacher-supported ChatGPT feedback, AI-supported peer feedback, and hybrid feedback suggest complementarity rather than simple replacement when the human layer anchors criteria, nuance, and evaluative authority (Bozorgian and Rahimi, 2025; Guo and Wang, 2024; Han and Li, 2024; Lee, 2024; Zhang et al., 2025). The proposed benefit depends on human guidance improving regulation of the AI layer; additional comments or teacher presence alone need not confer an advantage.

This model reframes design. Learning-oriented AI systems should not aim only to maximize correction accuracy or output polish. They should preserve authorial core processing. Strategy-focused LLM writing instruction and activity-theory design work support guided, reflective, locally adapted use rather than mere access to a powerful tool (Zhang and Jiang, 2026; Zhang Y. and Liu Y., 2025). Productive designs ask learners to attempt first, mark confidence, request focused feedback, compare alternatives, explain revisions, and complete transfer checks. The interface should not merely return better text. It should create occasions for learners to think with and against the feedback.

## 7 Hypotheses

The theory yields eight learner-process hypotheses. A separate methodological proposition (M1) is developed in the empirical agenda. Table 2 summarizes the hypotheses and their measurement logic; the cited findings motivate these predictions rather than establish the complete proposed pathways.

**Table 2:** Testable hypotheses and measurement logic for Calibration-Bypassing-Uptake theory.

ID	Core proposition	Key indicators	Predicted learning pattern
H1	Feedback quality predicts transfer through calibration, parallel regulation pathways, and strategic uptake in that order.	Separate source and self-calibration; time-ordered regulation measures; uptake decisions.	Ordered indirect paths predict transfer; direct and alternative paths remain testable.
H2	Unrestricted early AI use increases the gap between assisted draft quality and later less-supported writing.	Timing of AI access; presence or absence of self-generated draft; confidence marking; transfer-check completion.	Early unrestricted use improves the assisted draft but produces weaker delayed transfer than calibrated post-attempt use.
H3	Assistance directiveness has an inverted-U relation with delayed transfer.	Cue, partial-solution, and complete-replacement conditions; matched accuracy, time, and suggestion count; process and load measures.	Intermediate assistance outperforms lower and higher directiveness; the turning point lies within the tested range.
H4	Explanatory, contrastive, and question-driven feedback produces stronger transfer than direct correction or full reformulation.	Feedback type; in-episode explanation and diagnosis as process indicators; later similar-problem correction as an outcome.	Direct correction may yield larger immediate text improvement, but explanatory feedback yields stronger delayed transfer.
H5	Learners with stronger metacognitive calibration benefit more from AI feedback.	Reliance versus expert-rated advice quality; confidence versus proximal diagnostic correctness, separately from delayed transfer.	Appropriate reliance and accurate self-judgment predict stronger transfer.
H6a-c	Feedback literacy and calibration scaffolds separately moderate proficiency-related and anxiety-related bypassing risks.	Baseline proficiency, anxiety, and feedback literacy; randomized scaffolds; process coding; separate interaction terms.	Higher literacy and scaffolds attenuate each risk slope; no combined vulnerability score is assumed.
H7	Anxiety reduction and revision persistence support learning only when paired with strategic uptake.	Writing anxiety; enjoyment; persistence time; number and quality of revisions; explanation depth.	Reduced anxiety predicts transfer when it frees attention for monitoring and revision, but not when it encourages delegation.
H8	Calibration-focused human guidance improves source, genre, and authorship judgments under matched conditions.	Comparable AI access, feedback, and time; calibration accuracy; justified feedback decisions; strategic uptake.	Transfer benefits are mediated by improved processes; equivalent AI-only scaffolding may yield equal gains.

*Hypothesis 1*. Higher-quality generative AI feedback should predict delayed transfer through an ordered indirect pathway: dual calibration, then cognitive regulation and affective-motivational regulation as parallel pathways, then strategic uptake. Source calibration and self-calibration are assessed separately before processing; regulation is measured during processing and uptake at the feedback decision. Direct effects and competing orderings remain estimable rather than being assumed absent. This prediction extends feedback literacy accounts of regulated uptake (Carless and Boud, 2018; Liu and Deris, 2025).

*Hypothesis 2*. Unrestricted early AI use should produce a stronger dissociation between assisted draft quality and delayed less-supported writing than calibrated AI use. The dissociation should be largest when learners use AI before generating their own plan or draft, and smaller when they draft first, record confidence, receive effort-preserving feedback, and complete transfer checks (Darvishi et al., 2024; Yan et al., 2025).

*Hypothesis 3*. Holding feedback accuracy, target, exposure time, and suggestion count comparable, increasing assistance directiveness should have an inverted-U relationship with delayed transfer. Directiveness means how much of the target operation AI completes: a cue identifying a problem, a partial worked solution, or a complete replacement. Intermediate assistance should reduce extraneous demand while preserving learner processing; complete replacement should increase bypassing risk. Use frequency and generated word count are separate exposure measures, and suggestion overload is a separate manipulation. The predicted curve requires graded conditions spanning the range and evidence of a turning point within that range, rather than a quadratic term alone. Load and process measures should test the proposed mechanism (de Bruin et al., 2020; Yao and Fan, 2025).

*Hypothesis 4*. Explanatory, contrastive, and question-driven AI feedback should produce stronger delayed transfer than direct correction or full reformulation, even when direct correction produces larger immediate draft improvement. The predicted mediator is depth of processing during revision, measured through operation-specific explanations and error diagnosis; later correction of similar problems is a separate transfer outcome (Lukešová and Jennings, 2026).

*Hypothesis 5*. Learners with stronger metacognitive calibration should benefit more from AI feedback than learners with overtrust, undertrust, overconfidence, or underconfidence. Calibration should be measured by the alignment between reliance and expert-rated feedback quality, and between pre-response confidence and correctness on a separate proximal diagnostic task, not the delayed transfer outcome. The most successful AI users may not be those who trust AI most, but those who know where trust is warranted (Jacovi et al., 2021; Lee et al., 2025; Zhang et al., 2020).

*Hypothesis 6*. *H6a*: Lower baseline proficiency should predict more process-coded bypassing, with a weaker negative proficiency slope at higher baseline feedback literacy. *H6b*: Higher baseline writing anxiety should predict more bypassing, with a weaker positive anxiety slope at higher feedback literacy. *H6c*: Randomly assigned calibration scaffolds should attenuate each risk relationship separately, compared with otherwise comparable tasks without scaffolds. These predictions distinguish limited evaluation resources from anxiety-driven closure; they require separate interaction tests, not a combined vulnerability score or an assumed higher-order interaction. Proficiency, anxiety, and feedback literacy are measured before AI access (Carless and Winstone, 2023; Pearson, 2024; Shen and Tao, 2025).

*Hypothesis 7*. AI feedback should reduce writing anxiety and increase revision persistence when experienced as low threat and controllable. These affective benefits should predict durable learning only when coupled with strategic uptake. Reduced anxiety supports learning when it frees attention for monitoring; it undermines learning when it encourages wholesale delegation (Dewaele and MacIntyre, 2014; Wang and Wang, 2024; Zhang S. and Liu X., 2025).

*Hypothesis 8*. At comparable AI access, feedback content, and time on task, human guidance that explicitly supports source evaluation, genre judgment, and authorship decisions should improve calibration and justified feedback decisions relative to AI-only feedback. Any advantage in higher-order revision or delayed transfer is expected to operate through these changes and strategic uptake. No general superiority is predicted for human involvement that does not improve these processes; an AI-only condition providing equivalent calibration support may perform equally well (Han and Li, 2024; Yin, 2025; Zhang et al., 2025; Zou S. et al., 2026).

## 8 An empirical agenda for testing the theory

Testing the theory requires designs that can see both product and process. A minimal study should include baseline writing, AI-supported revision, and delayed less-supported writing. Baseline writing establishes current competence. Supported revision shows what AI helps learners produce. Delayed writing shows whether any part of the scaffolded operation has become more independent. Without all three, researchers cannot separate learning from performance support.

Methodological proposition M1. Assisted draft gains alone cannot identify durable learning and may overstate it when attributed to learner competence. Within the same intervention, researchers should compare baseline-adjusted immediate performance with delayed outcomes on matched tasks, rather than assume that differences between unrelated studies reflect measurement alone. Unaided, reduced-support, and selective AI-supported transfer should be reported separately. The size and direction of any discrepancy are empirical questions, not a universal claim that all immediate post-tests overestimate learning (Fan et al., 2025; Yan et al., 2025).

Experimental contrasts should move beyond AI versus no AI. More informative designs would compare unrestricted AI use with calibrated AI use, early access with post-attempt access, full reformulation with hint-based feedback, and AI-only feedback with hybrid teacher-AI feedback. A calibrated-use condition might require learners to draft first, mark confidence beside selected sentences, request focused AI feedback, explain accepted revisions, and complete a no-AI transfer task 1 week later. If both groups improve assisted drafts but only the calibrated group improves delayed writing, the theory gains support. Such designs would complement current intervention and design-based studies by identifying mechanisms, not simply tool effects (Fan et al., 2025; Liu Z. M. et al., 2026; Wiboolyasarin et al., 2024; Zhang and Jiang, 2026; Zhang Y. and Liu Y., 2025).

Process data should be central. Keystroke logs can show whether learners revise after reading feedback or replace large blocks at once. Prompt logs can distinguish requests for explanation, generation, evaluation, translation, and style smoothing. Screen recordings can reveal comparison behavior. Confidence ratings can be collected before and after feedback. Revision explanations can be coded for depth. Textual revisions can be coded as surface, sentence-level, discourse-level, or goal-level changes. Recent work on learner engagement, prompting, feedback seeking, and self-regulated behavior in AI-mediated writing shows that these data are necessary for interpreting what students actually do with feedback (Hwang H. et al., 2025; Hwang M. et al., 2025; Jia et al., 2026; Koltovskaia et al., 2024; Ma et al., 2026; Wang, 2024; Yan, 2025; Yan and Zhang, 2024).

A preliminary coding framework should use the feedback episode, from an identifiable suggestion or request to the learner’s decision, as its unit. Before coding, specify the target learning operation and its instructional relevance. Table 3 assigns provisional process codes using converging traces and an opportunity to demonstrate reasoning. Full-text acceptance is not sufficient evidence of bypassing, and requesting an explanation is not sufficient evidence of strategic uptake. A learner can accept a complete reformulation strategically by evaluating and reconstructing its rationale; an explanation request can remain unprocessed if the learner copies the answer without applying it.

**Table 3:** Preliminary process coding for feedback episodes.

Code	Process evidence	Decision rule
Strategic uptake	A substantive rationale plus observed comparison, adaptation, verification, or independent reconstruction of the target operation.	Acceptance, adaptation, or rejection can qualify. Judge the reasoning against the operation; verbal fluency alone is insufficient.
Cognitive bypassing	AI performs the target operation; the learner adopts it without substantive evaluation or reconstruction, corroborated by an accessible proximal probe.	Require delegation plus converging evidence of omitted processing despite an opportunity. Do not infer it from copying, speed, or weak delayed transfer alone.
Mixed episode	Initial delegation is followed by substantive evaluation or reconstruction before the episode ends.	Retain the sequence and code phases separately; do not label the learner globally as bypassing.
Indeterminate	Logs, recall, or an appropriate opportunity to demonstrate processing are missing or conflict.	Leave unclassified; silence or an unavailable explanation is not proof of absent thought.

Proximal probes should concern the current operation, permit demonstration or an explanation in a familiar language, and be distinguished from later transfer tasks. Record whether reasoning was spontaneous or elicited, because probes can themselves support learning. Two coders should independently apply a preregistered manual while blind to delayed outcomes, report agreement before adjudication, and pilot task-appropriate thresholds. Aggregate episode proportions with indeterminate cases reported separately. This framework is proposed, not validated: its reliability and discriminant validity require testing. Later ability to explain, adapt, or transfer must be used to test predictions, not to relabel earlier process codes.

Calibration measures require special care. Source calibration can be assessed by comparing learner reliance with independent expert ratings of AI feedback quality and genre fit. Self-calibration can compare pre-response confidence with correctness on brief, separate diagnostic items. Both should be collected before the delayed outcome and kept distinct from the indicators used to classify uptake or bypassing. Human-AI decision research offers measurement logic for distinguishing appropriate reliance from overreliance and underreliance (Lee et al., 2025; Zhang et al., 2020). Delayed transfer must not define the calibration mediator or the bypassing code that is then used to predict that same outcome.

Researchers should also code the target of each AI suggestion. A grammar correction, lexical replacement, stance adjustment, citation recommendation, paragraph reordering, and conceptual expansion do not impose the same demands. They differ in verifiability, risk, and learning value. Local grammar feedback may be checked against a rule, whereas a global argument suggestion requires disciplinary judgment and authorial intent. Coding feedback targets would allow studies to identify where AI most reliably scaffolds learning and where it most often invites bypassing.

Delayed transfer tasks should vary in distance, timing, and support. Near transfer can involve the same error type in new sentences, while farther transfer can require a new topic or genre. Across prespecified delays, unaided tasks assess internalized skill; reduced-support tasks permit bounded hints but restrict full generation; AI-supported tasks retain comparable access while testing selective reliance, detection of misleading suggestions, justified genre adaptation, and authorial control. Use matched baseline measures and task difficulty, document model and interface conditions, and report the three outcomes separately. Include unfamiliar or deliberately flawed suggestions when testing critical AI use so that higher scores cannot be explained by a more capable tool or routine prompting alone.

Qualitative work can deepen the model by examining appraisals. Interviews and stimulated recall can ask why learners accepted or rejected suggestions, whether they felt ownership over revisions, and how they judged reliability. Such data matter because cognitive bypassing is partly hidden. A learner may produce a polished draft and report limited understanding of the changes. Another may reject many suggestions for strong reasons. The second learner may look less compliant but more self-regulated. Mixed-methods studies of AI-generated feedback already show that learner explanations, perceptions, and engagement histories are indispensable for interpreting revision outcomes (Koltovskaia et al., 2024; Ma et al., 2026; Tossell et al., 2024; Wang and Wang, 2025).

Boundary conditions also need testing. Much evidence comes from higher education and Asian EFL contexts. Reviews of IDLE show that out-of-class digital English learning is context-dependent and especially salient in Asian EFL settings, where learner agency, digital literacies, access, and institutional expectations shape participation (Soyoof et al., 2023; Liu et al., 2025c). The theory should be examined across school stages, proficiency bands, genres, stakes, institutional AI policies, and teacher framing. Reviews of AI-mediated language learning show rapid expansion but uneven coverage across contexts, cognitive constructs, and task designs (Crosthwaite and Sun, 2026; Feng et al., 2025; Lee et al., 2026; Woo et al., 2024). The model may operate differently in timed exams, process-writing courses, remedial grammar instruction, English for academic purposes, and thesis writing because these contexts alter appraisals of cost, authorship, reliability, and controllability.

Ethical and assessment questions should be treated as psychological variables, not external add-ons. Policies about acceptable AI use shape anxiety, legitimacy beliefs, and authorship appraisals. Rubrics shape whether learners seek polish or learning. If final text quality is rewarded without process evidence, cognitive bypassing becomes rational. If assessment includes revision logs, explanations, and transfer tasks, strategic uptake becomes valuable. Critical digital-literacy and AI-policy work in L2 writing therefore belongs inside the learning ecology (Dang and Wang, 2024; Darvin, 2025; Liu et al., 2025b; Sandstead and Kibler, 2025; Wang and Wang, 2025).

## 9 Design principles for calibrated AI writing support

Although this is a theory paper, the model implies concrete design principles. The first is attempt before assistance. Learners should produce a sentence, outline, draft, or error diagnosis before receiving AI feedback. This creates a cognitive baseline. Without an attempt, learners and teachers cannot know what the learner could do independently, where help was needed, or what changed after feedback. Hint-based or clue-before-correction designs provide practical ways to preserve this baseline (Lukešová and Jennings, 2026).

The second principle is confidence before feedback. Learners should mark confidence in selected claims, sentences, or revision choices before AI responds. This converts vague uncertainty into metacognitive data. It also makes surprise educational. When a low-confidence sentence is correct, learners can adjust self-beliefs upward. When a high-confidence sentence contains an error, they can examine why monitoring failed. This principle follows from metacognitive monitoring and from human-AI decision research on confidence and reliance (Flavell, 1979; Zhang et al., 2020).

The third principle is local before global. AI should first provide focused feedback on learner-produced text rather than whole-text generation. Local feedback keeps attention near the learner’s own problem representation. Global generation is not always inappropriate, but it carries greater bypassing risk because it can replace planning, organization, and style decisions at once. For many learners, a small well-timed hint is more educational than a polished paragraph.

The fourth principle is to compare before accepting. Learners should compare original and revised versions, name the difference, and decide whether meaning has changed. This is especially important in academic writing because polish can subtly alter stance. Changing may suggest to demonstrate that, for example, can overstate evidence. Comparison protects voice and authorship by requiring learners to inspect whether revised text still expresses their intended claim (Mo and Crosthwaite, 2025; Sandstead and Kibler, 2025).

The fifth principle is explaining after revision. Learners should briefly explain why they accepted, adapted, or rejected AI feedback. The explanation need not be long; it needs to make reasoning visible. A note such as “accepted” because the revised subject matches the verb, or “rejected” because the model changed the causal claim, can convert revision into self-regulation. This aligns with feedback literacy research, which treats effective uptake as interpretive and evaluative rather than mechanical (Carless and Winstone, 2023; Lipnevich and Panadero, 2021).

The sixth principle is transfer after support. After AI-assisted revision, learners should complete a short unaided task requiring the same operation. If AI helps with hedging, the learner writes two new hedged claims. If AI helped with topic-sentence focus, the learner revises a new topic sentence without the model. Complementary tasks can assess bounded-support performance and selective AI use; these should not be substituted for an unaided-skill claim. The unaided check targets internal control and is compatible with learning research on retrieval and generation (Bjork and Bjork, 2020; Roediger and Karpicke, 2006).

The seventh principle is teacher framing. Teachers should present AI neither as a forbidden shortcut nor as an infallible tutor, but as a fallible feedback source requiring judgment. When teachers ask for revision rationales and discuss useful and misleading AI feedback, they normalize calibrated use. This is important because students’ choices between ChatGPT and teachers are shaped by perceived authority, convenience, metacognitive awareness of AI limits, and authorship concerns (Lee, 2024; Wang, 2024; Yin, 2025). These principles also support academic integrity. Authorship becomes visible through original attempts, AI suggestions, revision rationales, and transfer attempts rather than through final-product surveillance alone (Dang and Wang, 2024). By analogy, documentation demands should remain proportionate to their verification value (Lan, 2026).

In practice, these principles can be combined into a short learning cycle: draft, predict, ask, compare, explain, revise, and transfer. The cycle does not prohibit AI. It changes the unit of assessment from the final text alone to the learner’s regulatory decisions around the text. This matters for high-stakes writing courses because students will likely continue to encounter AI outside institutional control. The educational response should therefore cultivate judgment under AI-rich conditions rather than pretend that writing can be returned to a pre-AI environment.

## 10 Discussion

Generative AI changes EFL writing because it changes the relation among effort, confidence, performance, and competence. Older feedback environments often gave learners too little feedback or delivered it too late. AI-mediated environments may give learners more feedback than they can evaluate. Scarcity is replaced by abundance. Delay is replaced by immediacy. The central problem becomes regulation of guidance.

Calibration-Bypassing-Uptake theory argues that AI-mediated writing development depends on whether learners remain psychologically involved in the operations that build competence. Calibration explains how learners decide what to do with feedback. Cognitive bypassing explains why a strong product can emerge without strong learning. Strategic uptake explains how external guidance becomes internal regulation. Distinct delayed transfer outcomes test its consequences without determining the process codes. These mechanisms belong together because they describe one educational event from different angles.

The theory avoids two weak positions. One treats AI as a simple accelerator of learning and infers value from better drafts, higher motivation, or reduced anxiety. The other treats AI as an inherent threat and assumes that less AI means more competence. Both miss the conditional nature of learning. AI can scaffold or substitute. It can lower the harmful load or remove useful effort. It can support self-efficacy or inflate it. The difference depends on task design, learner knowledge, stakes, teacher framing, and calibration.

For educational psychology, the contribution is conceptual precision. AI is not the explanatory endpoint. It is a new arrangement of feedback, social presence, cognitive offloading, and metacognitive uncertainty. The stable psychological constructs remain familiar: working memory, self-regulation, monitoring accuracy, motivation, anxiety, enjoyment, self-efficacy, feedback engagement, and transfer. What is new is their configuration. Learners now regulate writing, while external intelligence can generate convincing language at any moment.

This precision also clarifies the practical stakes. If educators define success as a cleaner submitted draft, AI will appear overwhelmingly beneficial. If they define success as independent transfer, calibrated judgment, and accountable authorship, the evidence will be more differentiated. Some AI practices will remain valuable, especially those that make criteria explicit and support repeated revision. Others will need redesign because they reward learners for bypassing exactly the operations that writing education is meant to cultivate.

The model also changes how classroom innovation should be evaluated. A highly usable AI workflow may still be educationally weak if it eliminates prediction, explanation, and self-testing. Conversely, a slower workflow may be more valuable if it leaves a trace in learners’ later judgment. This point is especially relevant for EFL contexts where students may use AI to compensate for unequal access to feedback. Equity requires access to support, but access alone is not enough. Learners also need criteria for deciding when support is accurate, when it changes meaning, and when it should be converted into a transferable strategy.

The theory is also falsifiable. If unrestricted AI use produces the same delayed less-supported writing gains as calibrated use, the bypassing claim weakens. If full reformulation produces stronger transfer than contrastive explanation after controlling for baseline ability, the feedback-type hypothesis needs revision. If calibration measures do not mediate AI effects on learning, calibrated trust may be less central than proposed. A useful theory should risk being wrong in informative ways.

The construct itself would be challenged if independently coded bypassing cannot be distinguished reliably from offloading, shallow processing, automation overreliance, or reduced agency, or adds no explanatory value beyond those measures and baseline ability. Its proposed learning cost would be challenged by precise, replicated evidence that process-confirmed bypassing yields equivalent or better transfer than strategic uptake under matched task and support conditions. The proposed ordering would be challenged if repeated time-resolved measurements and targeted interventions consistently favor uptake preceding calibration, or parallel and reverse-order models over the specified indirect paths. Calibration interventions should change subsequent regulation and uptake if calibration has the proposed role. These tests require successful manipulations, reliable measures, and adequate precision; a nonsignificant association alone is not decisive. Evidence against bypassing, its predicted consequence, or path ordering would call for revision of the corresponding claim rather than *post hoc* reclassification of episodes.

Several limitations follow. The model focuses on EFL writing, not all language learning. Speaking, reading, vocabulary learning, and disciplinary writing may involve different forms of calibration and bypassing. A legal-AI bibliometric study highlights semantic complexity and ethical concerns (Lan and Zhang, 2026). The account is also more developed for university learners because much current evidence is concentrated there. Younger learners may need different scaffolds, while professional writers may use AI with stronger prior criteria. The model treats less-supported writing as one valuable outcome alongside selective AI-supported transfer; these capacities may develop unevenly. Even if future workplaces normalize AI-supported writing, independent judgment remains necessary because users must decide whether output is accurate, ethical, relevant, and their own.

AI-mediated writing will keep changing as interfaces become more adaptive and multimodal. Yet the core educational question will remain: does the learner become better able to think, plan, revise, and judge, or only better able to obtain fluent text? Apparent fluency is not the enemy. It becomes educationally limited when it outruns understanding. Durable learning begins where learners can turn feedback into reasons, reasons into revision, and revision into future control.

## Data Availability

The original contributions presented in the study are included in the article/supplementary material, further inquiries can be directed to the corresponding author.
